# HPV *E6/E7* RNA *In Situ* Hybridization Signal Patterns as Biomarkers of Three-Tier Cervical Intraepithelial Neoplasia Grade

**DOI:** 10.1371/journal.pone.0091142

**Published:** 2014-03-13

**Authors:** Mark F. Evans, Zhihua Peng, Kelli M. Clark, Christine S.-C. Adamson, Xiao-Jun Ma, Xingyong Wu, Hongwei Wang, Yuling Luo, Kumarasen Cooper

**Affiliations:** 1 Department of Pathology, University of Vermont, Burlington, Vermont, United States of America; 2 Department of Pathology and Laboratory Medicine, Fletcher Allen Health Care, Burlington, Vermont, United States of America; 3 Advanced Cell Diagnostics, Inc., Hayward, California, United States of America; The Chinese University of Hong Kong, Hong Kong

## Abstract

Cervical lesion grading is critical for effective patient management. A three-tier classification (cervical intraepithelial neoplasia [CIN] grade 1, 2 or 3) based on H&E slide review is widely used. However, for reasons of considerable inter-observer variation in CIN grade assignment and for want of a biomarker validating a three-fold stratification, *CAP-ASCCP LAST* consensus guidelines recommend a two-tier system: low- or high-grade squamous intraepithelial lesions (LSIL or HSIL). In this study, high-risk HPV *E6/E7* and *p16* mRNA expression patterns in eighty-six CIN lesions were investigated by RNAscope chromogenic *in situ* hybridization (CISH). Specimens were also screened by immunohistochemistry for p16^INK4a^ (clone E6H4), and by tyramide-based CISH for HPV DNA. HPV genotyping was performed by GP5+/6+ PCR combined with cycle-sequencing. Abundant high-risk HPV RNA CISH signals were detected in 26/32 (81.3%) CIN 1, 22/22 (100%) CIN 2 and in 32/32 (100%) CIN 3 lesions. CIN 1 staining patterns were typified (67.7% specimens) by abundant diffusely staining nuclei in the upper epithelial layers; CIN 2 lesions mostly (66.7%) showed a combination of superficial diffuse-stained nuclei and multiple dot-like nuclear and cytoplasmic signals throughout the epithelium; CIN 3 lesions were characterized (87.5%) by multiple dot-like nuclear and cytoplasmic signals throughout the epithelial thickness and absence/scarcity of diffusely staining nuclei (trend across CIN grades: P<0.0001). These data are consistent with productive phase HPV infections exemplifying CIN 1, transformative phase infections CIN 3, whereas CIN 2 shows both productive and transformative phase elements. Three-tier data correlation was not found for the other assays examined. The dual discernment of diffuse and/or dot-like signals together with the assay’s high sensitivity for HPV support the use of HPV *E6/E7* RNA CISH as an adjunct test for deciding lesion grade when CIN 2 grading may be beneficial (e.g. among young women) or when ‘LSIL vs. HSIL’ assignment is equivocal.

## Introduction

The management of patients with pre-invasive cervical lesions is dependent on the assigned histological classification. There is considerable debate over the terminology that best facilitates patient care and consensus usage between pathologists [1]–[6]. Lesion misdiagnosis may result in over-treatment of patients by way of unnecessary loop electrosurgical excision procedure (LEEP) or similar interventions that damage the cervix and that can result in impaired fertility or other cervical health-related problems as well as the anxiety, discomfort, inconvenience and cost engendered [3], [7]–[9]. Misdiagnosis leading to under-treatment might result in patient presentation at a later date with invasive disease. Diagnostic accuracy and agreement among histopathologists is therefore critical. A three-tier grading of neoplastic lesions as cervical intraepithelial neoplasia (CIN) grades 1, 2, or 3 is widely used. However, considerable inter-observer variation in the judgment of dysplastic status and CIN grade has been shown by many studies [1]–[6]. The distinctions between CIN mimics and CIN and between CIN 1 and CIN 2+ can be challenging: atrophic changes, inflammation, pregnancy and angle of biopsy sampling can all complicate histopathologic assessment [1]–[6]. The recently published *CAP-ASCCP Lower Anogenital Squamous Terminology Standardization (LAST)* consensus guidelines have therefore recommended a two-tier approach in which lesions are divided as low- or high-grade squamous intraepithelial lesions (LSIL or HSIL) [5], [6]. This recommendation is made on the basis that current biomarkers (hematoxylin and eosin [H&E] staining or p16^INK4a^ immunohistochemistry [IHC]) do not support a standardized three-tier system.

In this study we have investigated high-risk (HR) HPV *E6/E7* mRNA chromogenic *in situ* hybridization (CISH) signal patterns as markers of CIN/SIL grade using a novel detection assay (RNAscope, Advanced Cell Diagnostics, Hayward, CA) [10]. The sensitive detection of mRNA by non-isotopic CISH has been problematic and robust IHC assays for HPV E6 or E7 are unavailable. The RNAscope HR 18 HPV assay is designed to detect *E6/E7* RNA for eighteen different HR HPV genotypes (HPVs 16, 18, 26, 31, 33, 35, 39, 45, 51, 52, 53, 56, 58, 59, 66, 68, 73, 82). The test utilizes 10 pairs of oligonucleotide probes per HPV type. Each oligo-probe has a 5′ ∼25 base region that binds specifically with an *E6* or *E7* sequence and the probe pairs are designed to hybridize contiguously. At the 3′-end of each probe in the pair is a non-HPV hybridizing 14 base sequence: the resulting 28 base sequence hybridizes with the 5′-prime end of ‘pre-amplifier’ oligonucleotides subsequent to the initial HPV hybridization step. Signal amplification is achieved by the sequential hybridization of amplifier sequences that bind to the pre-amplifiers and label-probes conjugated with horseradish peroxidase (HRP) that bind the amplifiers. The theoretical amplification for a given HPV type is 4,000-fold. The primary ‘cooperative’ hybridization step that requires contiguous dual probe binding in order for successful pre-amplifier hybridization helps ensure assay specificity; the use of ∼25 base HPV specific probes supports hybridization to mRNA in formalin-fixed, paraffin-embedded (FFPE) specimens, in which mRNA is typically relatively degraded. Additionally, our investigation of HPV *E6/E7* RNA expression has been complemented with the examination of *p16* mRNA CISH (RNAscope) expression patterns, HPV DNA CISH signal patterns, p16^INK4a^ IHC staining profiles and HPV DNA genotyping of lesions. Using these combined tests, the aim was to investigate whether distinct HPV-related biological profiles underlie CIN categories and whether RNAscope assays may contribute to CIN/SIL grading.

The causative role of HR HPV genotypes in the development of CIN and cervical carcinoma is well established [11]. The likely primary site of HPV infection is transformation zone basal cells accessed via micro-abrasions in the epithelial layer. A latent phase infection, in which low-copy number HPV episomes are maintained simply by cell division, shows minimal pathologic features. The productive or permissive phase of infection may show koilocytic and CIN 1 morphologic changes typified by basaloid type cells in the lower third of the epithelium. In this phase, the HPV life-cycle is coupled to epithelial differentiation and maturation. HPV DNA episome amplification and capsid production occurs in mid-to superficial epithelial layers and infective HPV virions are released as cells slough off from the epithelial surface. A persistent infection may give rise to the transformative phase characterized by dysregulation of the regular HPV life-cycle and loss of cellular differentiation and maturation resulting in high-grade CIN morphology characterized by basaloid type cell involvement up to the middle to two thirds portion of the lesion (CIN 2) or the full thickness (CIN 3) [1], [12]–[16].

The HR-HPV *E6* and *E7* oncogenes have transformative properties and impact a wide variety of cellular targets; the best described are degradation of the p53 tumor suppressor protein by E6 protein and abrogation of pRB tumor suppressor protein functioning by E7 protein [10]–[14]. Chronic expression of E6 and E7 oncoproteins may lead to the acquisition of genomic instability and proliferative capacity [12]–[16]. Latent phase HPV infections show undetectable *E6/E7* expression and low levels of HPV DNA replication; productive phase lesions display low-level *E6/E7* transcription in the lower third to half of the epithelium and high levels of HPV DNA replication; and, transformative infections demonstrate high levels of E6/E7 expression throughout the full thickness of the epithelium and low levels of HPV DNA replication. E6/E7 over-expression may result by abrogation of the *E2* gene, which is involved in *E6/E7* regulation, by integration of HPV DNA into the host cell genome that typically involves physical disruption of the *E2* gene, or, from *E2* silencing by methylation or other epigenetic events [12]–[17].

p16^INK4a^ immunohistochemistry (IHC) is commonly used for distinguishing reactive from neoplastic atypias and for assisting in ≥CIN 2 designation. The p16^INK4a^ protein acts as a regulator of cell cycle control: high levels of p16^INK4a^ (expressed in response to genotoxic damage, aging, or disturbances to cellular differentiation/maturation or oncogenes) normally prevent S-phase progression by inhibition of cyclin-dependent kinases (CDKs); this results in hypophosphorylated pRB that binds E2F transcription factors. HR HPV E7 protein disrupts pRB binding of E2F: unbound E2F stimulates S-phase progression; p16^INK4a^ over-expression is the observed consequence as E2F also stimulates p16^INK4a^ expression, which no longer acts in a feedback loop to inhibit its own expression [14], [15]. Histopathologic correlation (two-tier) between the degree of epithelial layer p16^INK4a^ staining and CIN grade and/or latent/productive/transformative phase of infection has been widely reported. Whilst HR-HPV status has clinical utility as a ≥CIN 2 risk biomarker for cervical cytology screening [18], HPV genotyping does not support histopathologic lesion grading [19]. Rather, histologic categorization requires biomarkers co-equivalent with the neoplastic grade of the lesion and its potential for progression to invasive disease.

## Materials and Methods

### Ethics

This study was conducted with the approval of the University of Vermont (UVM) Institutional Review Board (IRB) Committee on Human Research in the Medical Sciences (CHRMS). The requirement for written informed patient consent was waived by the committee for reasons including the use of archival specimens collected >2 years prior to study initiation (in accordance with College of American Pathologists [CAP] guidelines) and from patients who had received the standard of care required for the treatment of CIN.

### Specimens

Eighty-six CIN lesions collected from eighty-three patients were identified by electronic record search (CoPath Plus, Cerner Corporation, Kansas City, MO): CIN 1 (n = 32), CIN 2 (n = 22), and CIN 3 (n = 32 [2 specimens had concurrent microinvasion]). Lesions were graded on the basis of the original clinical sign-out diagnosis, together with H&E review (KC and KMC) of sections cut from formalin-fixed, paraffin-embedded (FFPE) tissue blocks. Cases were then coded and all subsequent assays were performed masked to the CIN grade assignment. Morphologically normal epithelia adjacent to CIN lesions and/or in separate tissues co-located in an FFPE block were used for internal negative control test purposes. Patient ages ranged from 17 to 57 years (mean 28.8, median 27.0, SD 7.9).

### HPV Genotyping

A ribbon (∼5–10 sections) of FFPE cervical tissues was cut into a 1.5 mL microfuge tube; the microtome blade was wiped with xylene-substitute and DNA Away (Molecular BioProducts, San Diego, CA) to prevent sample cross-contamination. Tissues were treated with several washes with xylene to remove paraffin-wax and then rehydrated by vortex mixing with 100% and 70% ethanol. Air dried tissues were digested overnight with proteinase K (400 µg/ml in 50 mM NaCl, 10 mM Tris-Cl pH 8.0 at 50°C) and purified DNA was recovered using a column clean-up method (DNeasy Tissue kit, Qiagen, Valencia, CA). DNA was quantified using a NanoDrop 2000 spectrophotometer (NanoDrop, Wilmington, DE). PCR for HPV L1 sequences was performed on 100 ng DNA using GP5+/6+ primers as previously described [20]. Briefly, 100 ng of extracted DNA was subject to 50 cycles of PCR in reaction combined with 1 µM each primer, 3.5 mM MgCl_2_, 200 µM ACGU dNTP mix (Fermentas), 0.2 units HotStaTaq and 1X HotStaTaq buffer (Qiagen, Valencia, CA), 1 unit uracil *N*-glycosylase (UNG) (Epicentre Biotechnologies, Inc., Madison, WI). The reaction mixture was incubated at 37°C for 30 minutes then heated to 95°C for 15 minutes followed by cycles 94°C for one minute, annealing for 1.5 minutes and extension at 72°C for 2 minutes; a touchdown annealing approach was adopted beginning with a temperature of 55°C and decreasing by 0.5°C per PCR cycle until a temperature of 40°C was reached. The detection of a ∼150 base pair amplicon was taken as evidence of HPV positive status. Specific HPV type identity was determined by cycle sequencing of the purified PCR amplicon and NCBI BLAST search. Positive control PCR was performed using PC03/05 primers for the detection of a 209 base pair β-globin sequence [21].

### RNA Chromogenic *In Situ* Hybridization

HR 18 HPV *E6/E7* CISH: The RNAscope 2.0 assay was performed according to supplier instructions (Advanced Cell Diagnostics, Hayward, CA) using a proprietary probe combination capable of detecting eighteen different HR HPV types. Briefly, 5 µm slide-mounted sections were de-paraffinized through 100% xylene and ethanol washes. Tissues were then treated serially with: Pre-Treatment 1 solution (endogenous hydrogen peroxidase block with Pretreat 1 solution for 10 minutes at room temperature); Pre-Treatment 2 (100°C, 15 minutes immersion in Pretreat 2 solution); and, Pre-Treatment 3 (protease digestion, 40°C for 30 minutes); rinses with water were performed after each Pre-Treatment step. Tissues were then hybridized in HR 18 HPV hybridization solution, without a cover slip, at 40°C for 2 hours in a HybEZ Oven (Advanced Cell Diagnostics, Hayward, CA). After wash buffer steps, signal amplification from the hybridized probe was performed by the serial application of Amp 1 (PreAmplifier step), Amp 2 (signal enhancer step), Amp 3 (amplifier step), Amp 4 (Label Probe step), Amp 5 and Amp 6 (signal amplifications steps); wash buffer steps were performed after each Amp step. HRP activity was then demonstrated by the application of 3,3′-diaminobenzidine (DAB) for 10 minutes at ambient temperature. Sections were then counterstained with hematoxylin, dehydrated through graded ethanol (50%, 70% and 100%) and xylene and then mounted with Cytoseal XYL (Thermo Fisher Scientific Inc., Waltham, MA). Staining data was recorded according to thickness of epithelial staining, presence and amount of diffuse, and/or punctate nuclear staining and cytoplasmic staining as well as signal intensity.

*p16* CISH: CISH for *p16* mRNA expression was performed as above using a proprietary oligoprobe combination for *p16* mRNA sequences. Staining was recorded in terms of thickness of epithelial staining and intensity of nuclear/cytoplasmic signals.

### RNA Chromogenic *In Situ* Hybridization Control Assays

RNAscope CISH positive and negative tests was performed for every specimen using proprietary probes for human sequence *ubiquitin C* (to demonstrate detectable mRNA in the FFPE samples) and *Bacillus subtilis dapB* mRNA targets respectively. *Ubiquitin C* staining was scored to confirm the presence of cytoplasmic signal and intensity was also noted. *Bacillus subtilis dapB* staining was reviewed to confirm absence of staining.

RNA target hybridization assays: Validation of hybridization with mRNA sequences was investigated on a limited set of specimens by tissue pretreatments with RNase A (QIAGEN Inc., Valencia, CA) 10 mg/mL in PBS, 30 minutes 40°C) and with DNase I (Sigma-Aldrich Corp., St. Louis, MO) 80 µg/mL in 10 mM TrisCl, 2.5 mM MgCl_2,_ 0.5 mM CaCl_2,_ pH7.5, 30 minutes 40°C). Additionally, samples were hybridized with HPV sense sequence probes that should be expected not to yield HPV mRNA signals.

Specimen control assays: Positive control samples included cervical carcinomas and CIN lesions previously established as HPV positive by GP5+/6+ PCR in conjunction with HPV DNA CISH. Negative control samples include normal cervical epithelium demonstrated as HPV negative by PCR and CISH.

### HPV DNA Chromogenic *In Situ* Hybridization

HPV DNA CISH was performed as previously described [22]. Briefly, 5 µm slide-mounted sections were de-paraffinized etc. as above and then immersed in 10 mM sodium citrate pH 6.0 for 40 minutes at 95°C followed by a 20 minutes cool down period. After treatment with 3% hydrogen peroxide in phoshphate buffered saline for 10 minutes at room temperature, tissues were digested with 100 µg pepsin (Sigma P-7012, Sigma, St. Louis, MO) in 0.2 M HCl for 5–15 minutes. Specimens were hybridized with HPV Wide Spectrum biotinylated probe (Dako Cytomation, Inc., Carpentaria, CA [that detects HPV types 6, 11, 16, 18, 31, 33, 35, 39, 45, 51, and 52]) overnight after co-denaturation of probe and tissue DNA, by heating at 93°C for eight minutes and cooling to 37°C in a Vysis Hybrite (Abbot Molecular, Des Moines, Il). After performing stringency washes (0.1 SSC, 50°C, 3×10 minutes), hybridized probe was detected by tyramide signal amplification (GenPoint Catalyzed Signal Amplification System, Dako Cytomation Inc, Carpentaria, CA) and visualized using 3-aminoethylcarbazole (AEC) substrate. CISH was performed minus HPV probe in the hybridization mix as a negative control. Positive controls included previously verified HPV positive cervical carcinomas. Staining data was recorded according to thickness of epithelial staining, and presence and amount of diffuse and/or punctate nuclear staining as previously described. Diffuse signals are indicative of episomal HPV and punctate signals can indicate HPV integrated into the cell genome [23].

### p16^INK4a^ Immunohistochemistry

IHC for p16^INK4a^ was performed using clone E6H4 (CINtec Histology kit, mtm laboratories, Westborough, MA) according to kit instructions: following de-paraffinization and rehydration, slides were immersed in epitope retrieval solution at 95–99°C for 10 minutes followed by a 20 minutes cool down period. Peroxidase-blocking reagent was then applied for 5 minutes after which mouse anti-human p16^INK4a^ was applied for 30 minutes followed by visualization reagent for 30 minutes and finally 3,3′-diaminobenzidine (DAB) tetrachloride substrate. Negative control assays were performed using reagents as provided in the CINtec Histology kit. Staining was recorded with reference to thickness of epithelial staining, intensity (0 [negative], 1+ [weak], 2+ [medium], 3+ [strong]) and nuclear or cytoplasmic signal.

### Statistical Analyses

Statistical analyses were performed using GraphPad InStat software (GraphPad Software, Inc. La Jolla, CA).

## Results

### HPV Genotyping

All CIN samples tested positive for β-globin demonstrating amplifiable quality DNA was extracted from the specimens. HPV was detected in 30/32 (93.8%) CIN 1 lesions: high-risk (HR) types were found in 27/32 (84.4%) and low-risk (LR) types were identified in 3/32 (9.4%) samples (Table 1). Twenty of 22 (90.9%) CIN 2 lesions tested HPV positive (all HR, Table 1). Thirty-one of 32 CIN3 (96.9%) tested HPV positive (all HR, Table 1). HPV 16 detection increased with CIN grade (P<0.0001 [Chi-Squared Test for Trend]). A wider range of HPV types was detected among CIN 1 lesions (n = 13) than among CIN 2 (n = 5) or CIN 3 (n = 3) (Table 1).

**Table 1:** HPV genotype distribution amongst the CIN 1, CIN 2 and CIN 3 study specimens.

	CIN 1 (n = 32)	CIN 2 (n = 22)	CIN 3 (n = 32)
**HPV positive**	30 (93.8%)	20 (90.9%)	31 (96.9%)
**HPV 16**	10 (31.3%)	15 (68.2%)	29 (90.6%)
**HPV 31**	6 (18.8%)	2 (9.1%)	1 (3.1%)
**HPV 35**	2 (6.3%)	1 (4.6%)	–
**HPV 39**	2 (6.3%)	–	–
**HPV 45**	1 (3.1%)	–	–
**HPV 51**	1 (3.1%)	1 (4.6%)	–
**HPV 51**	1 (3.1%)	–	–
**HPV 56**	1 (3.1%)	1 (4.6%)	1 (3.1%)
**HPV 58**	1 (3.1%)	–	–
**HPV 66**	1 (3.1%)	–	–
**HPV 73**	1 (3.1%)	–	–
**HPV 6**	1 (3.1%)	–	–
**HPV 11**	2 (6.3%)	–	–

### Staining Data Summary

**Figure 1:**
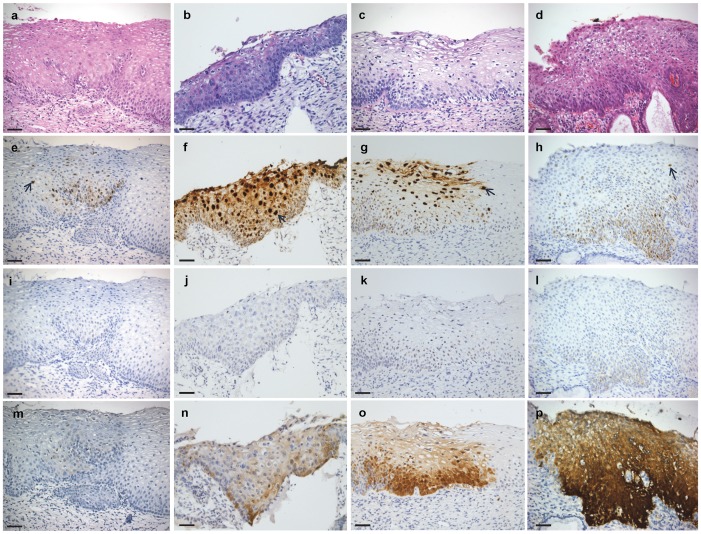
Representative CIN 1 staining patterns. Columns a-d: Four H&E stained CIN 1 specimens (a, b, d, HPV 16 positive; c, HPV 35 positive); row e-h: HPV *E6/E7* RNA CISH staining patterns; row i-l: *p16* mRNA CISH staining patterns; row m-p: p16^INK4a^ IHC staining patterns. The first specimen (a) may represent an early lesion displaying low-level HPV staining (e) mostly in the lower half of the lesion with occasional superficial cells showing diffusely stained nuclei (black arrows, e-h). *p16* mRNA signals were undetected. p16^INK4a^ staining was scored as negative. The second specimen (b) shows a productive phase HPV staining pattern as indicated by the abundant diffusely stained nuclei throughout the epithelial thickness (f). *p16* mRNA signals were barely detectable (j). p16^INK4a^ staining shows patchy positivity (n). Specimen c also shows a productive phase pattern of HPV expression (g). *p16* mRNA signals were detectable in the lower third of the epithelium (k) matching p16^INK4a^ IHC staining (o). Most CIN 1 lesions showed the staining patterns shown for specimens b, f, j, n and c, g, k, o. An exception to this staining trend was found in specimen d, which showed strong staining for HPV in the lower part of the lesion and occasional diffuse staining nuclei (h). *p16* mRNA signals were detectable in the lower epithelial layers and strong p16^INK4a^ staining was detected throughout the lesion; possibly, this specimen represents a CIN lesion entering into a transformative phase directly from CIN 1 morphology. All images were originally taken using a 20X objective lens. Scale bar: 50 µm.

**Figure 2:**
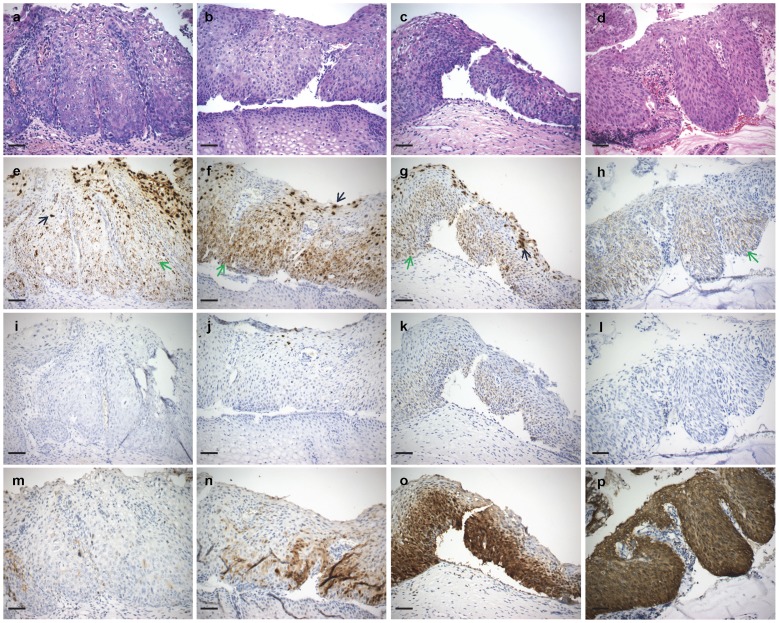
Representative CIN 2 staining patterns. Columns a-d: Four H&E stained CIN 2 specimens (a, c, d, HPV 16 positive; b HPV 35 positive); e-h: HPV *E6/E7* RNA CISH staining patterns; i-l: *p16* mRNA CISH staining patterns; m-p: p16^INK4a^ IHC staining patterns. The first three specimens (columns a, b, c) show typical CIN 2 HPV *E6/E7* staining as a mixture of superficial layer abundant diffusely staining cell nuclei (black arrows) together with strong nuclear and cytoplasmic dot-like signals in the lower layer through to the surface (green arrows). *p16* mRNA staining was variable from negative (i) to medium (k); some superficial diffusely stained nuclei were noted (k). p16^INK4a^ staining ranged from negative (m) to two-thirds thickness (o). The fourth case (column d) represents an exception to typical CIN 2 staining patterns. No diffuse staining was detected (h) and p16^INK4a^ staining was strong throughout the epithelial layer. The specimen may represent a candidate for reassignment as CIN 3. All images were originally taken using a 20X objective lens. Scale bar: 50 µm.

**Figure 3:**
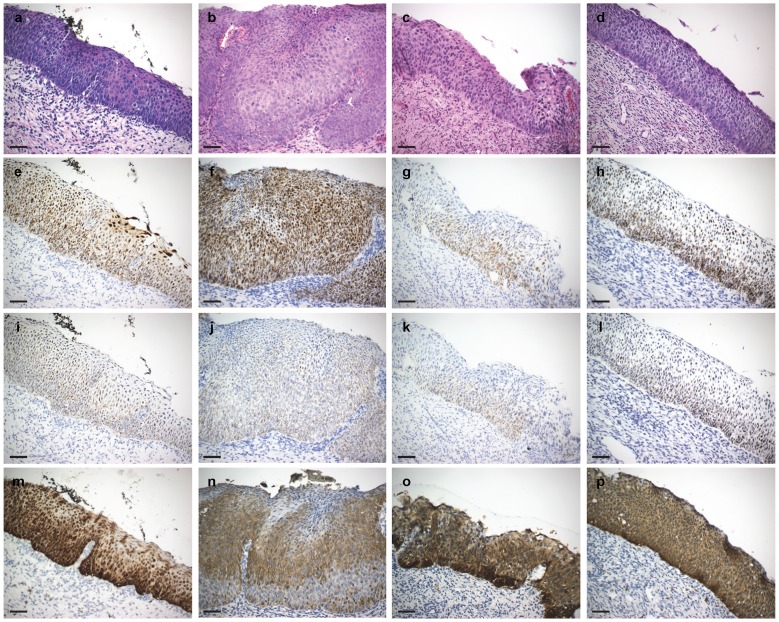
Representative CIN 3 staining patterns. Columns a-d: Four H&E stained CIN 3 specimens (all HPV 16 positive); e-h: HPV *E6/E7* RNA CISH staining patterns; i-l: *p16* mRNA CISH staining patterns; m-p: p16^INK4a^ IHC staining patterns. All CIN 3 lesions showed staining patterns consistent with the transformative phase of HPV infection. HPV staining was detected throughout the thickness of the epithelium as strongly staining nuclear and cytoplasmic dots. Occasional diffusely staining nuclei were detected in some lesions in the outermost superficial layer; focal ‘CIN 2 patterns’ were noted along with some CIN 3 (e). *p16* mRNA staining was notable qualitatively stronger and detectable in all layers (I, j, k, l). All images were originally taken using a 20X objective lens. Scale bar: 50 µm.

CISH and IHC data from the study are summarized in Table 2 and in Figures 1–3 (and Figures S1–S3). In Table 2, staining descriptions were simplified given the limited numbers of specimens per CIN grade and are reported with reference to staining predominantly basal up to mid-epithelial layers (≤BME), basal up to full epithelial-thickness (BME+), and for CISH, the detection in the superficial layer of diffuse nuclear signals (SupD). For the RNAscope assays, ≤BME and BME+ layers data refers to the detection of nuclear and cytoplasmic dot-like signals and the SupD layer data refers to the detection of diffusely stained whole nuclei. For the HPV DNA CISH assay, ≤BME and BME+ layers data refers to the detection of punctate signals and the SupD layer data refers to the detection of diffusely stained whole nuclei. The p16^INK4a^ IHC staining presented as diffuse nuclear and cytoplasmic staining as indicated (Table 2).

**Table 2:** Summary of CIN staining patterns of high-risk HPV RNA and *p16* mRNA by RNAscope CISH, p16^INK4a^ IHC and HPV DNA CISH.

	CIN 1	CIN 2	CIN 3	P-value*
**HR HPV RNA CISH**				
Positive	26/32 (81.3%)	22/22 (100%)	32/32 (100%)	**0.004**
≤BME	6/31 (19.4%)	1/22 (4.5%)	0/32 (0%)	**0.005**
BME+	19/31 (61.3%)	21/22 (95.5%)	32/32 (100%)	**<0.0001**
SupD	21/31 (67.7%)	14/22 (63.6%)	2/32 (6.3%)	**<0.0001**
***p16*** ** mRNA CISH**				
Positive	27/31 (87.1%)	21/22 (95.5%)	32/32 (100%)	**0.030**
≤BME	14/27 (45.2%)	4/22 (18.2%)	0/32 (0%)	**<0.0001**
BME+	12/27 (38.7%)	17/22 (77.3%)	32/32 (100%)	**<0.0001**
**p16^INK4a^ IHC**				
Positive	29/32 (90.6%)	21/22 (95.5%)	30/31 (96.8%)	0.300
≤BME	24/31 (77.4%)	3/22 (13.6%)	0/31 (0%)	**<0.0001**
BME+	4/31 (12.9%)	18/22 (81.8%)	30/31 (96.8%)	**<0.0001**
**HPV DNA CISH**				
Positive	10/29 (34.5%)	13/20 (65.0%)	15/29 (51.7%)	0.200
≤BME	1/29 (3.4%)	0/20 (0%)	2/29 (6.9%)	0.500
BME+	3/29 (10.3%)	8/20 (40.0%)	13/29 (44.8%)	**0.004**
SupD	6/29 (20.7%)	8/20 (40.0%)	4/29 (13.8%)	0.750

≤BME: staining confined to basal through to mid-epithelial layers; BME+: staining extending up to the surface epithelium; SupD: superficial layer diffusely stained nuclei detected.

*Chi-Square Test for Trend.

### HPV *E6/E7* RNA CISH Staining and CIN Grade

Eighty of 86 (93.0%) CINs tested positive for HPV by RNA CISH. HPV was detected in 26/32 (81.3%) CIN 1 s. The six negatives consisted of three LR HPV samples (one HPV-6 and two HPV-11) and three HR infections (two HPV-16 and one HPV-31). The 26 positives were all from specimens infected with HR HPV types. The epithelium from one specimen was thin and partially denuded and was discounted from further descriptive review. Most (21/31 [67.7%]) CIN 1 lesions were characterized by the presence of intense diffusely stained nuclei mostly in the top 1/3-1/2 of the epithelium with weak to strong intensity dot-like nuclear and cytoplasmic signals mainly in the lower layers (Figure 1). Four (12.9%) CIN 1 s showed dot-like signals only and no diffusely staining nuclei: in two of these nuclear and cytoplasmic staining was confined to the lower 1/2 of the epithelium (Figure 1); in the other two specimens signals were detectable at all levels of the epithelium, nuclear and cytoplasmic.

All 22 CIN 2 lesions stained positive for HPV: one (4.5%) showed only nuclear and cytoplasmic dot-like signals confined to the lower 1/2 of the epithelium; 21/22 (95.5%) were characterized by medium to strong nuclear and cytoplasmic dot-like signals throughout the epithelial thickness (Figure 2); 14 (66.7%) of these specimens also showed medium to strong diffuse nuclear signals in the upper 1/2 to 1/3 of the epithelial layer.

HPV was detectable in all 32 CIN 3 lesions. CIN 3 s mostly (28/32 [87.5%]) presented with full-thickness epithelial layer staining with medium to strong nuclear and cytoplasmic dot-like signals (Figure 3); in four (12.5%) samples, the staining was confined to the lower 2/3 of the epithelium. Two (6.3%) CIN 3 s examined showed occasional superficial diffuse nuclear staining.

Significant trends (Table 2) were noted across CIN grades 1–3 with reference to numbers of specimens staining positive for HR HPV, proportion of epithelium showing staining, and the presence of diffusely stained cell nuclei in the superficial layers. In particular, the presence of diffusely stained nuclei in the superficial layer detected by RNAscope was significantly higher in CIN 2 s (63.6%) than that in CIN 3 s (6.3%) (p<0.0001).

### HPV DNA CISH Staining and CIN Grade

Eight of the initial 86 specimens selected for study were discounted from DNA CISH review; four specimens were un-interpretable due to high-background staining and in the other four samples the lesion was no longer present. HPV was detected by biotinyl-tyramide-based CISH in 38/78 (48.7%) CIN lesions (vs. 80/86 [93.0%] RNAscope HR HPV [P<0.0001]). Additionally, in contrast to RNAscope, HPV DNA CISH signals were frequently focal and sporadic, whereas the RNAscope signals were profuse and detected in a high percentage of cells within a lesion.

Ten (34.5%) of 29 CIN 1 specimens were DNA CISH positive: 5 (20.7%) showed superficial layer diffuse nuclear staining only, one (3.4%) showed superficial diffuse nuclear signals and sporadic punctate signals at all levels of the epithelium. Four (13.8%) specimens showed only punctate signals: 1 (3.4%) showed relatively abundant strong signals in the lower two thirds of the epithelium, 1 sporadic weak signals in the lower two thirds, 1 sporadic weak signals in the lower half of the epithelium, and one sample showed frequent strong punctate signals in the lower portion of the epithelium but the superficial layers missing. Of the CIN 2 s, 13/20 (65.0%) were positive: superficial diffuse nuclear staining was detected in 8 (40.0%) samples, of which 3 (15.0%) also showed sporadic punctate signals at all levels of the epithelium and 1 showed superficial punctates (not indicated in Table 2); 5 (25.0%) samples showed only punctate signals; these were focal and sporadic and detectable in the lower two-thirds to full thickness of the epithelium. Among the CIN 3 s, 15/29 (51.7%) tested positive. Specimens with punctate (all epithelial levels) and diffuse (superficial) signals were noted in 4 (13.8%) samples. Punctate signals only were detected in 11 (37.9%) lesions: in 9 instances the staining was throughout the epithelial layers, in 2 in the lower half and in one in the superficial layers only. Punctate signals detectable in ≥ lower 1/2 of the epithelium were significantly more common in high-grade CIN than CIN 1 (Table 2). Three CIN lesions were positive for HPV types not included in the wide spectrum probe cocktail (one CIN 1− HPV 66, one CIN 2–HPV66 and one CIN 3– HPV56) and may have tested CISH negative for this reason. Examples of DNA CISH staining are included as Figure S4.

### *p16* mRNA CISH Staining and CIN Grade

CISH *p16* signals were detected in 78/85 (91.8%) CIN lesions; signals were predominantly cytoplasmic in all positive samples. Among CIN 1 s, *p16* expression was detected in 24/30 (80.0%) specimens; (1/32 specimen excluded for high background staining and one for loss of lesion). Staining was confined to the lower 1/3–1/2 of the epithelium in 11/30 (36.7%) samples; l0 were weak and 1 medium in staining intensity. Thirteen (43.3%) samples showed signals in the lower 2/3 to full epithelial thickness; 10 weak and 3 medium in intensity. Twenty-one (95.5%) of 22 CIN 2 s demonstrated *p16* staining. In four (18.2%) specimens signals (all weak cytoplasmic) were confined to the lower 1/3 to 1/2 of the epithelium and in 17 (77.3%) samples staining was present in the lower 2/3 to full epithelial thickness (6 cases weak; 10 cases medium; 1 case strong). All 32 CIN 3 specimens showed lower 2/3 (9 [28.1%]) to full thickness (23 [71.9%]) epithelial staining. Signals were weak in 10, medium in 10 and strong in 12 specimens. *p16* expression detectable in basal up to and including superficial layers increased with CIN grade (Table 2, Figures 1–3 and S1–S3).

### p16^INK4a^ IHC and CIN Grade

p16^INK4a^ IHC was performed on 85 CIN lesions; one case was discounted through loss of the lesion in the course of sectioning. Staining (nuclear and cytoplasmic) was detected in 80/85 (94.1%) of specimens. Of 32 CIN 1 specimens, 29 (90.6%) showed staining; a thin epithelial layer was noted in one samples and discounted from further descriptive analysis. Twenty-four (77.4%) of 31 samples demonstrated lower 1/3 −1/2 staining; weak intensity in 7, moderate in 8, and strong in 9 specimens. Four (12.9%) samples showed lower 2/3 to 3/3 epithelial thickness staining that was moderate to strong in intensity. p16^INK4a^ staining was found in 21/22 (95.5%) CIN 2 specimens: in 3 (13.6%) samples confined to the lower 1/3 −1/2 (weak, medium and strong intensity); in 18 (81.8%) cases, 2/3–3/3 moderate to strong epithelial staining was noted. One CIN 3 sample was discounted from the study through loss of the lesion. Of 31 CIN 3 s, 30 (96.8%) showed 2/3-full thickness epithelial staining that was moderate to strong in intensity; one samples was negative. p16^INK4a^ staining patterns were in general distinguished CIN 2+ for CIN 1 but did not support the differentiation of CIN2 from CIN3 (Table 2, Figure 1–3 and S1–S3).

### RNA CISH Positive and Negative Control Assays

*Ubiquitin C* (*UBC*) mRNA expression was detectable in all specimens as abundant moderate to intense cytoplasmic signals demonstrating the preservation of RNAscope detectable mRNA in the FFPE specimens. Staining with the *Bacillus subtilis dapB* mRNA probe was noted in several samples (mainly CIN 1 s) as fine/weak dot-like signals in all tissue compartments: this was interpreted as non-specific assay-related staining (Figures S5 and S6); allowance was made for this in interpreting HPV *E6/E7* or *p16* signals (readily distinguishable in terms of much greater intensity and abundance and association with a lesion). [Technical note: further to this study a newer version (v2.0) of the RNAscope assay has been marketed showing a cleaner background with the *dapB* probe.].

### Validation of HPV RNA Detection

The RNAscope RNA assay has been developed for the sensitive demonstration of mRNA by *in situ* hybridization. To confirm this application in the detection of HPV infections, a few selected tissues were pretreated with RNase A or DNase I prior to hybridization with antisense and sense-strand HPV probes. DNase I exposure confirmed that HPV mRNA signals are detectable in epithelial cell cytoplasm and nuclei (Figures S5 and S6); intense diffuse nuclear signals were also detectable in some instances, especially CIN 1 and CIN 2 lesions (Figures S5 and S6). RNase A digestion resulted in the loss of cytoplasmic signals and the majority of nuclear signals that were present as single to multiple dot-like signals; however, some strong nuclear staining (especially diffuse signals) remained though diminished in intensity compared to RNase A pretreated samples (Figures S5 and S6). CISH with the HR HPV sense probe resulted in the detection of nuclear signals suggesting hybridization with DNA as the probe should be non-complementary to transcribed HPV sequences (Figures S5 and S6). These data indicate that the HR HPV RNAscope CISH assay may also detect some HPV DNA when the HPV DNA load is high as in cells containing productive phase HPV (see Discussion). RNase A pretreatment of samples hybridized for *Ubiquitin C* mRNA detection resulted in the non-detection of any signal (Figures S5 and S6).

## Discussion

In this study, lesions graded as CIN 1, 2 or 3 after routine H&E review were investigated by PCR for HPV genotype infection, by CISH for HPV DNA, HPV *E6/E7* RNA and *p16* mRNA *in situ* staining patterns, and by IHC for p16^INK4a^ expression. The major findings (Table 3) are threefold. Firstly, the data show that the RNAscope HR HPV RNA CISH assay is a highly sensitive and specific method for the direct confirmation by bright-field microscopy of an HPV infection in a lesion. Secondly, the assay supports the inference of different phases of an HPV infection and thirdly, the data are suggestive of a biological basis for CIN 1, 2 and 3 histopathology by showing a concordance between CIN grade and HPV phase of infection; as such HPV RNA CISH may be a valuable adjunct test for use in CIN grading.

**Table 3:** Summary of major findings.

	Take Home Points
**1)**	**HR HPV RNA CISH is a potential gold standard method for oncogenic ** ***E6/E7*** ** expression detection.**
	89.7% HR HPV positive CIN 1 lesions and 100% HR HPV positive CIN 2 and CIN 3 showed staining.
	CISH signals are easily observable at low magnification and resemble IHC staining.
	Tailored assay conditions may be unrequired: one common protocol worked well for all study specimens.
	The assay is automatable.
**2)**	**HPV RNA expression patterns are consistent with models of HPV infective biology.**
	HPV productive phase infection is indicated by mid- to superficial layer diffusely-stained nuclei.
	Transformative phase infection is shown by multiple dot-like signals throughout the epithelium.
**3)**	**HPV RNA signal patterns correlate with three-tier CIN lesion grading.**
	CIN 1 is typified by productive phase CISH signal patterns.
	CIN 2 lesions show an admixture of productive and transformative signal pattern features.
	CIN 3 lesions characterized by transformative staining patterns.

With respect to staining effectiveness, 26 (89.7%) of 29 HR HPV positive CIN 1 lesions and 100% of 22 CIN 2 and 32 CIN 3 s stained positive by HPV RNA CISH. CISH staining in lesions was readily identifiable: within the epithelial stratum showing CISH signals ≥50% cells stained; moderate to strong signal intensity was noted in nearly all cases; staining was judged as weak in 4 (13.8%) CIN1 s, 1 (4.6%) CIN2 s and 1 (3.1%) CIN3 s. Staining was absent from morphologically normal tissues. This high level of HPV detection and the staining patterns detected (intense nuclear and cytoplasmic dots as well as diffusely staining nuclei) directly parallels data obtained from HPV RNA *in situ* hybridization assays performed on cell lines, CIN lesions and carcinomas using ^3^H, ^32^P or ^125^I-labelled riboprobes [24]–[31]. Possibly some signals, especially intense diffusely staining nuclei may represent the co-detection of RNA and DNA (Figures S5 and S6). This might be due to probe hybridization with ‘unzipped’ single-stranded HPV occurring during up-regulated HPV DNA episome synthesis in the productive phase of the HPV life cycle.

Tyramide-based CISH for HPV DNA was significantly less effective at staining CIN lesions than RNAscope (Table 1). In a previous tyramide-CISH study in our lab a much higher proportion of CINs stained for HPV DNA [22]. In that study we customized pre-treatment conditions (e.g. pepsin digestion time) for each specimen, prepared our own nick-translated labeled HPV probes and used a different version of tyramide staining kit [22]. However, even relative to that study, RNAscope HPV CISH signals show much more abundant and intense staining within a lesion. Additionally, non-specific staining was much less of a problem with the RNAscope than with the tyramide-based detection system.

These data support the use of HPV RNA CISH as a gold standard assay for determining whether an anogenital specimen (or even a head and neck tumor) is high-risk HPV positive and actively expressing oncogenic *E6/E7* transcripts. HPV RNA CISH may also be helpful for distinguishing normal/CIN mimics (such as immature squamous metaplasia, atrophy, inflammation/reactive changes, tangential sectioning artifacts) from true CIN. Clearly, overtreatment may result if non-CIN is mistaken as CIN and under-treatment when CIN goes unrecognized [32]. The RNAscope assay has been adapted for use on the Ventana DISCOVERY XT/ULTRA automated ISH platform supporting its potential for routine screening of specimens for HPV.

**Figure 4:**
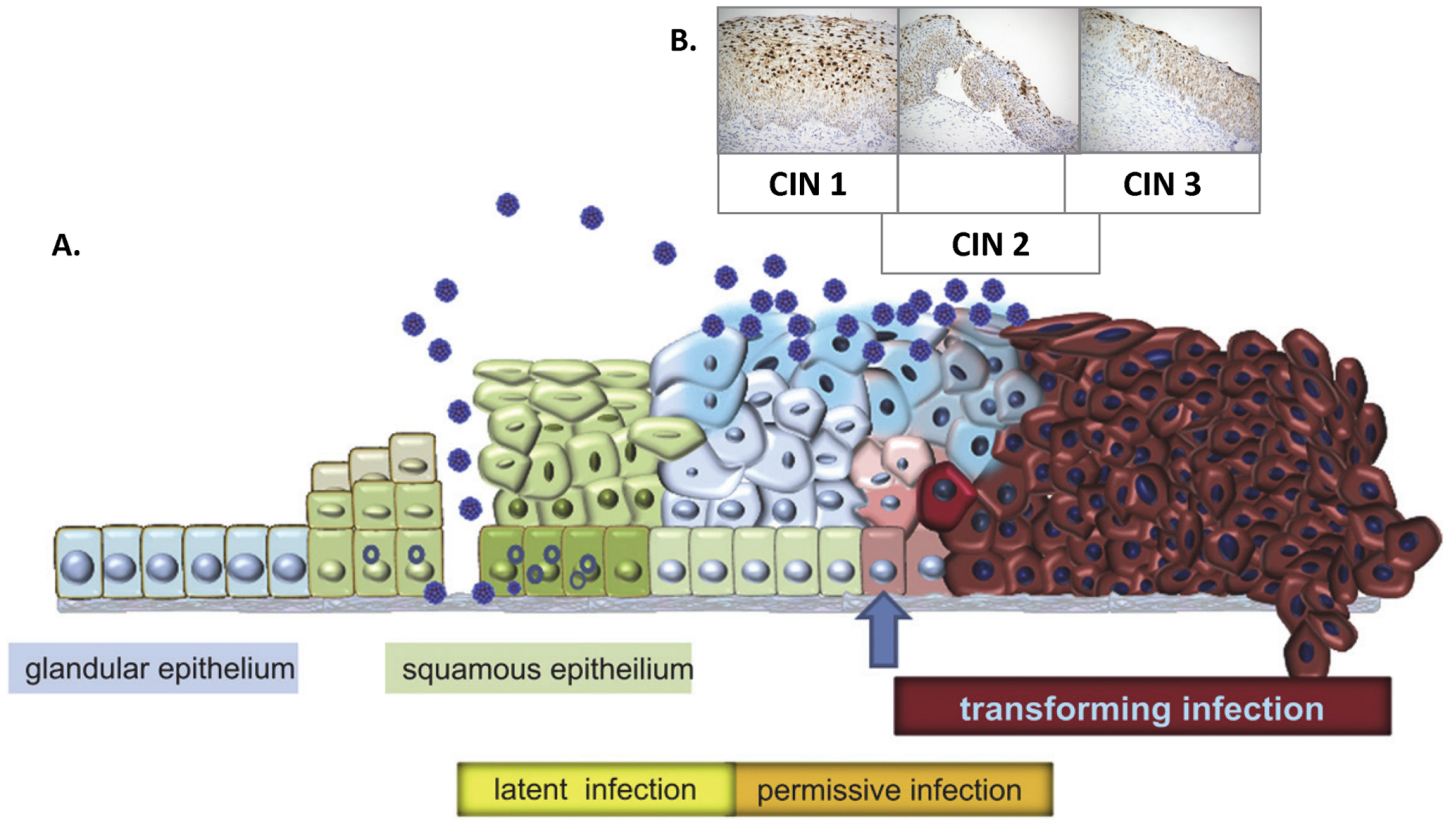
Correspondence of HPV *E6/E7* RNA staining patterns with HPV infective biology. **A.** HPV infection of basal cells occurring via micro-abrasions to the cervical epithelium may give rise to latent and subsequently productive (permissive) infections. The transforming phase is associated with upregulated *E6* and *E7* expression Blue arrow). **B.** The data from the present study suggests HPV *E6* and *E7* RNA expression detected by chromogenic *in situ* hybridization corresponds with HPV infective mode which in turn correlates with CIN grade. [Image A. (references 15 and 16) is reproduced with kind permission courtesy of Professor Magnus von Knebel Deobervitz, University of Heidelberg, Germany.].

The HPV RNA CISH staining patterns can be interpreted as markers of HPV phase of infection (Figure 4): latent, productive and transformative and the data from this study are suggestive that these patterns show a correlation with the three-tier CIN grading system. In particular, CIN 2 lesions demonstrated a distinctive staining pattern different from that CIN 1 s or CIN 3 s (Figures 1–3 and S1–S3; Table 2). CIN lesion grading by histopathological review of an H&E stained slide and the biological basis for CIN 1, 2 and 3 categories are controversial. The validity of CIN 2 as a distinctive classification has been questioned for reasons such as lack of diagnostic consensus and reproducibility, and as being nothing more than a convenience for equivocation [1]–[6]. If so then CIN grade is best divided as low-grade and high-grade [5], [6]. Arguments in favor of CIN 2 include its utility in deciding LEEP referrals especially when agreed upon by two pathologists [1]–[3]. There may also be a particular benefit in assigning the CIN 2 diagnosis for women younger than 25 years of age: 62–70% of these patients may show lesion regression to normal suggesting the possibility of conservative management [33], [34]. That CIN 1, 2 and 3 each represent distinct biological entities has also been suggested by multinomial regression model analyses, which have shown distinctive covariate profiles associated with each grade; for example, ‘baseline ASCUS cervical cytology’ and ‘ever been a smoker’ are associated with CIN 2 [35]. The RNAscope HPV staining pattern may be the first biomarker supporting the biological basis of CIN 2.

The CIN 1 cohort analyzed in this study was characterized by infection (93.8% of samples) with a relatively heterogeneous group (n = 13) of mainly HR HPV types. Just over a third of these infections were detectable by HPV DNA CISH and sixty percent of these included the detection of abundant diffuse staining cells in the superficial layers. HR HPV mRNA CISH showed signals in 81.3% of the specimens with superficial signals in two-thirds of cases. *p16* mRNA was detected by CISH in 87.1% of the lesions and extended past basal-mid (BME+) layers in 38.7% cases whereas p16 protein expression was detected in 90.6% of cases and was detected in BME+ layers in 12.9% of specimens. These findings are consistent with CIN 1 morphology harboring several HPV related biologies. Firstly, latent/early or possibly regressing lesions showing a low viral load and an absence of productive phase HPV replication (Figure 1, column a); secondly, and most usually, productive phase infections indicated by abundant superficial diffuse nuclear CISH staining patterns (Figure 1, columns b and c); and thirdly, lesions that may have the potential to progress as indicated especially by BME+ p16 protein and transformative mRNA expression (Figure 1, column d). These data are consistent with the finding that CIN 1 preceded by ASCUS or LSIL cytology are at low-risk of progression and may be treated conservatively [36]. Interestingly, De Marchi Triglia *et al.* have shown that basal layer HPV DNA CISH punctate signals are associated with CIN 1 lesions at risk for progression [37].

Among the 22 CIN 2, HPV DNA was detected by tyramide-CISH in 65.0% of specimens with superficial diffusely staining nuclei noted in 40.0% of samples, whereas RNAscope HPV signals were detected in 100% of specimens and superficial diffuse signals were noted in 68.2% of the specimens (Figure 2, columns a-c). *p16* mRNA and protein expressions were detectable through the greater part of the epithelial thickness in most cases. Of the remaining CIN 2 s, 27.3% showed only abundant intense full-epithelial thickness dot-like signals consistent with a transformative phase of HPV infection (Figure 2, column d). One (4.5%) specimen showed dot-like signals confined to the lower half of the epithelium; speculatively this might indicate a lesion in regression. These findings are consistent with the majority of CIN 2 lesions displaying a combination of both productive and transformative phases of HPV infection. Possibly, CIN 2 lesions found to be in transition to a transformed phase are appropriate for CIN 3 management, whereas CIN 2 lesions showing a productive phase pattern could be managed more conservatively.

All 32 CIN 3 lesions were HPV positive by RNA CISH: abundant intense nuclear and cytoplasmic dot-like signals were evident through the full thickness of the epithelium in 87.5% of these samples (Figure 3, columns a, b, d) and three (9.4%) showed concurrent focal/sporadic superficial diffusely staining nuclei; 9.4% samples showed abundant dot-like signals in the lower two-thirds of the epithelium and no superficial signals (Figure 3, column c). By HPV DNA CISH, 55.2% of CIN3 s were positive. Signals were less abundant than with the RNAscope assay and superficial diffuse signals were concurrent in four (13.8%) samples. *p16* mRNA expression was detected in all cases in two-thirds to full epithelial thickness. Signals were less abundant and intense than with the HPV mRNA assay. p16^INK4a^ IHC staining was abundant and detectable in two-thirds to full epithelial thickness apart from one specimen that was negative. These data support a view of CIN 3 being coequal with the transformative phase of HPV infection.

In summary, the findings from this study support the potential of RNAscope HPV RNA chromogenic in *situ* hybridization as an aid for confirming HPV infections in pathological specimens and for use in CIN histological grading. Although this study is limited by the numbers of CIN specimens assessed, the correlation between HPV RNA CISH signal patterns and CIN grade, and in particular the distinction between CIN 2 and CIN 3 is notable and worthy of further investigation; p16^INK4a^ IHC staining can indicate high-grade CIN but not the difference between CIN 2 and CIN 3 [16]. The data also suggest an alignment of CIN grade with biology: CIN 3 lesions can be defined in terms of the transformative phase HPV infection, CIN 2 s as containing elements of both transformative and productive phases and CIN 1 s as tissues that are mostly in the productive phase of HPV infection or low-abundance infections that may be nascent or regressing lesions. The recently published *CAP-ASCCP LAST* consensus guidelines for the standardization of terminology for HPV-associated lesions recommend a two-tiered nomenclature [5], [6]. The rationale for this is that no biomarker data support a 3-tier system and that CIN 2 has no biologic correlate and is “an intermediate category thought to represent a mixture of low-grade and precancerous disease that cannot be reliably distinguished based on hematoxylin and eosin (H&E) morphology” and that the histologic category is non-reproducible. However, it was conceded that the CIN 2 category might be retained as a qualifying notation in parentheses along with a 2-tiered classification to allay concerns that overtreatment might result from a strictly dichotomous grading approach [5], [6]. The data from this study do indeed support the notion that CIN 2 contains an admixture of low-grade (productive phase HPV) and precancerous disease (transformative phase HPV) but suggest this admixture could be reliably and reproducibly indicated on the basis of HPV *E6/E7* RNA CISH signal patterns. The sensitive and specific dual discernment of productive and transformative phase biomarkers by RNA HPV CISH supports its use for qualitative three-tier CIN grading or for discriminating ‘LSIL’ or ‘HSIL’ among indefinite cases when applying a two-tier system. Clearly, larger case control studies using a consensus panel of CINs are required to confirm and extend the empirical observations correlating high-risk HPV RNA CISH signal patterns with lesion grade. Additional studies are also required to assess the relationship of CISH signal patterns to CIN lesion potential for progression or regression.

## Supporting Information

Figure S1 **CIN 1 staining (expanded data).** Top row: H&E; second row: HPV *E6/E7* RNA CISH; third row: *p16* RNA CISH; fourth row: p16^INK4a^ IHC; fifth row: *UBC* RNA CISH; sixth row: *E.Coli dap B* RNA CISH. Column groups a-d (see Figure 1 legend). Column 1: CIN 1 lesion (HPV 31 positive) showing condylomatous features. Column 2: CIN 1 (HPV 31 positive) exception showing absence of productive phase HPV expression and strong p16^INK4a^ IHC staining through the lesion (note: epithelium is partially denuded). All images were originally taken using a 20X objective lens. Scale bar: 50 µm.
(PDF)

Figure S2 **CIN 2 staining (expanded data).** Top row: H&E; second row: HPV *E6/E7* RNA CISH; third row: *p16* RNA CISH; fourth row: p16^INK4a^ IHC; fifth row: *UBC* RNA CISH; sixth row: *E.Coli dap B* RNA CISH. Columns a-d (see Figure 2 legend for details). Column 1: CIN 2 lesion (HPV 16 positive) typical staining pattern. Column 2: CIN 2 (HPV 16 positive) exception showing limited productive phase HPV expression but negative for p16^INK4a^ IHC staining; lesion remained IHC negative on repeat staining. All images were originally taken using 20X objective lens. Scale bar: 50 µm.
(PDF)

Figure S3 **CIN 3 staining (expanded data).** Top row: H&E; second row: HPV *E6/E7* RNA CISH; third row: *p16* RNA CISH; fourth row: p16^INK4a^ IHC; fifth row: *UBC* RNA CISH; sixth row: *E.Coli dap B* RNA CISH. Columns a-d (see Figure 3 legend). Columns 1 & 2: HPV 16 positive lesions showing CIN 3 staining patterns; p16^INK4a^ IHC staining was negative in one instance (S7). All images were originally taken using 20X objective lens. Scale bar: 50 µm.
(PDF)

Figure S4 **HPV DNA CISH. a.** CIN 1 lesion superficial diffuse staining nuclei. **b.** CIN 2 lesion superficial diffuse staining nuclei. **c.** CIN 3 lesion showing diffuse and punctate signals through the thickness of the lesion. All images were originally taken using 20X objective lens. Scale bar: 50 µm.
(PDF)

Figure S5 **HPV** ***E6/E7*** **RNA CISH control tests: productive phase HPV 16 infection. a.** HPV diffuse nuclear staining signal patterns and fine nuclear and cytoplasmic dot-like signals after hybridization with antisense probe. **b.** HPV diffuse nuclear staining signal patterns after hybridization with sense-strand *E6/E7* probe. These indicate that some diffuse nuclear staining may be HPV DNA. **c.** Reduced HPV diffuse nuclear staining signal patterns and absence of fine nuclear and cytoplasmic dot-like signals after hybridization with antisense probe on tissues pretreated with RNase A. These data suggest diffuse nuclear staining is (partially) due to hybridization with HPV DNA and that the fine nuclear and cytoplasmic signals are HPV RNA. **d.** HPV diffuse nuclear staining signal patterns after hybridization with sense-strand HPV probe on tissues pretreated with RNase A suggestive of hybridization with HPV DNA. **e.** Absence of HPV staining signal patterns after hybridization with anti-sense-strand HPV probe on tissues pretreated with DNase I confirming diffuse signal patterns involve probe hybridization with DNA. **f.** Reduced intensity diffuse HPV staining signal patterns after hybridization with sense-strand HPV probe on tissues pretreated with DNase I confirming diffuse signal patterns involve probe hybridization with DNA. Detected signals may be due to incomplete DNase digestion of abundant productive phase HPV DNA. **g. & h.** Abundant *UBC* RNA staining (g) eliminated (h) after tissue pretreatment with RNase A. All images were originally taken using 40X objective lens. Scale bar: 20 µm.
(PDF)

Figure S6 **HPV** ***E6/E7*** **RNA CISH control tests: transformative phase HPV 16 infection. a.** HPV nuclear and cytoplasmic dot-like signals after hybridization with antisense probe. **b.** Absence of staining after hybridization with sense-strand HPV probe. This finding shows that the signals detected in (a) represent HPV RNA targets and not DNA. **c. & d.** Absence of any staining with anti-sense or sense-stand HPV probes on tissues pretreated with RNase A indicating the non-detection of HPV DNA targets. **e.** HPV positive staining after hybridization with antisense HPV probe on tissues pretreated with DNase I confirming signal patterns result from probe hybridization with RNA. **f.** Absence of staining in DNase I treated tissue hybridized with sense-strand HPV probe confirming that the anti-sense probes detect HPV *E6/E7* RNA. **g. & h.** Abundant *UBC* RNA staining (g) eliminated (h) after tissue pretreatment with RNase A. All images were originally taken using 40X objective lens. Scale bar: 20 µm.
(PDF)
