# Supplementary figures and images for: HPV E6/E7 RNA In Situ Hybridization Signal Patterns as Biomarkers of Three-Tier Cervical Intraepithelial Neoplasia Grade
Source: PLoS One. 2014 Mar 13;9(3):e91142. doi: 10.1371/journal.pone.0091142 (PMC3953338; doi:10.1371/journal.pone.0091142)

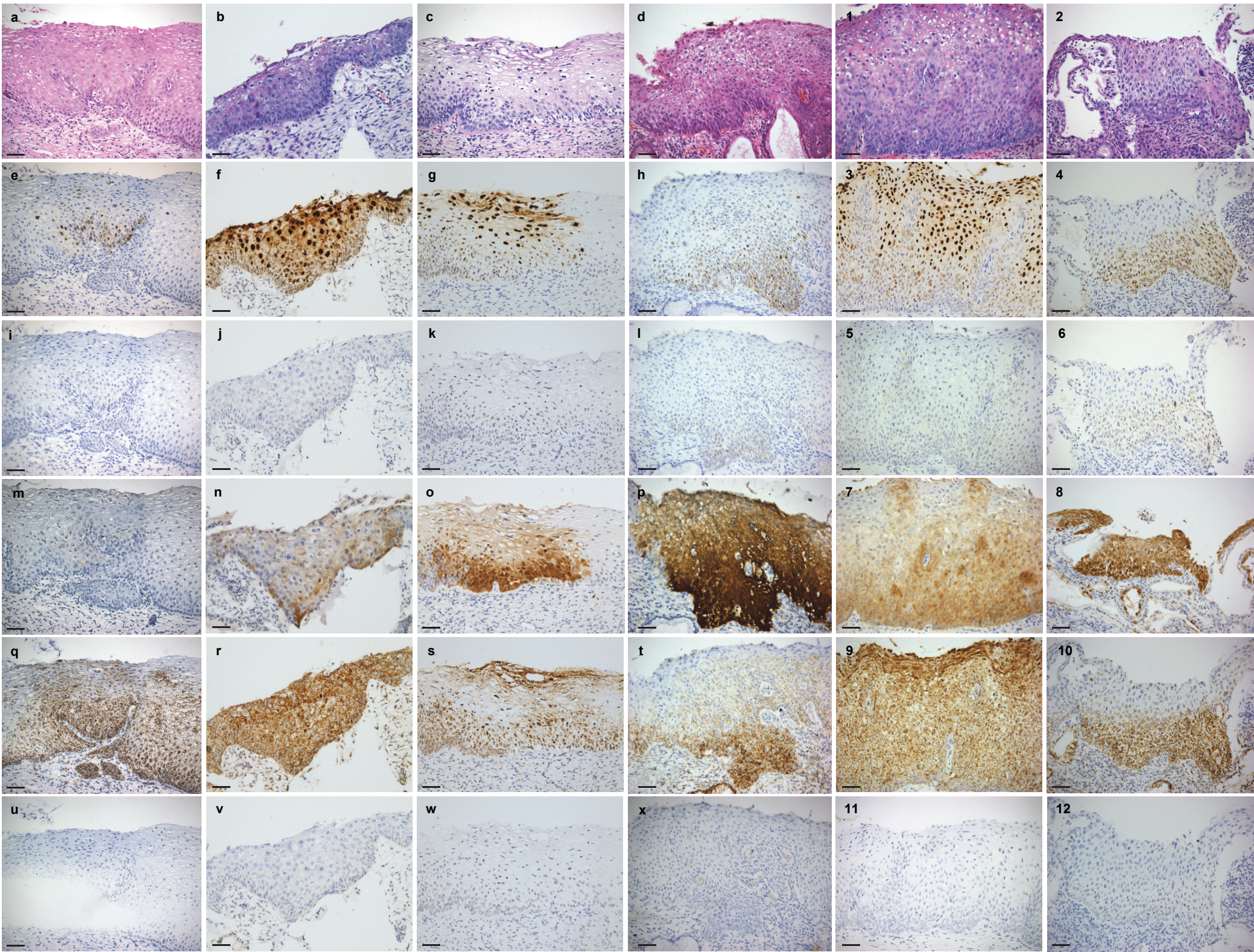

Supplement: Figure S1 — CIN 1 staining (expanded data). Top row: H&E; second row: HPV E6/E7 RNA CISH; third row: p16 RNA CISH; fourth row: p16INK4a IHC; fifth row: UBC RNA CISH; sixth row: E.Coli dap B RNA CISH. Column groups a-d (see Figure 1 legend). Column 1: CIN 1 lesion (HPV 31 positive) showing condylomatous features. Column 2: CIN 1 (HPV 31 positive) exception showing absence of productive phase HPV expression and strong p16INK4a IHC staining through the lesion (note: epithelium is partially denuded). All images were originally taken using a 20X objective lens. Scale bar: 50 µm. (PDF) [file pone.0091142.s001.pdf]

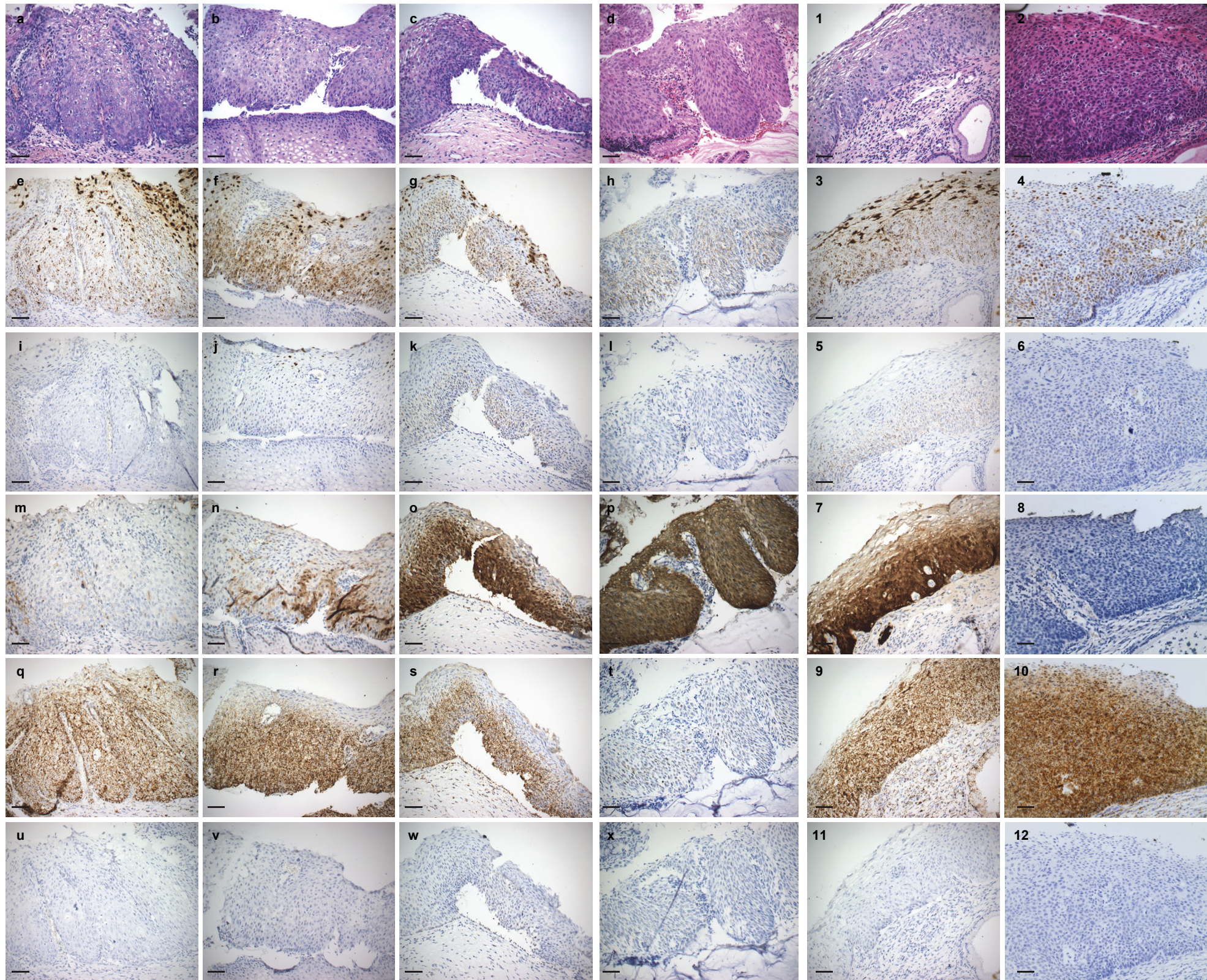

Supplement: Figure S2 — CIN 2 staining (expanded data). Top row: H&E; second row: HPV E6/E7 RNA CISH; third row: p16 RNA CISH; fourth row: p16INK4a IHC; fifth row: UBC RNA CISH; sixth row: E.Coli dap B RNA CISH. Columns a-d (see Figure 2 legend for details). Column 1: CIN 2 lesion (HPV 16 positive) typical staining pattern. Column 2: CIN 2 (HPV 16 positive) exception showing limited productive phase HPV expression but negative for p16INK4a IHC staining; lesion remained IHC negative on repeat staining. All images were originally taken using 20X objective lens. Scale bar: 50 µm. (PDF) [file pone.0091142.s002.pdf]

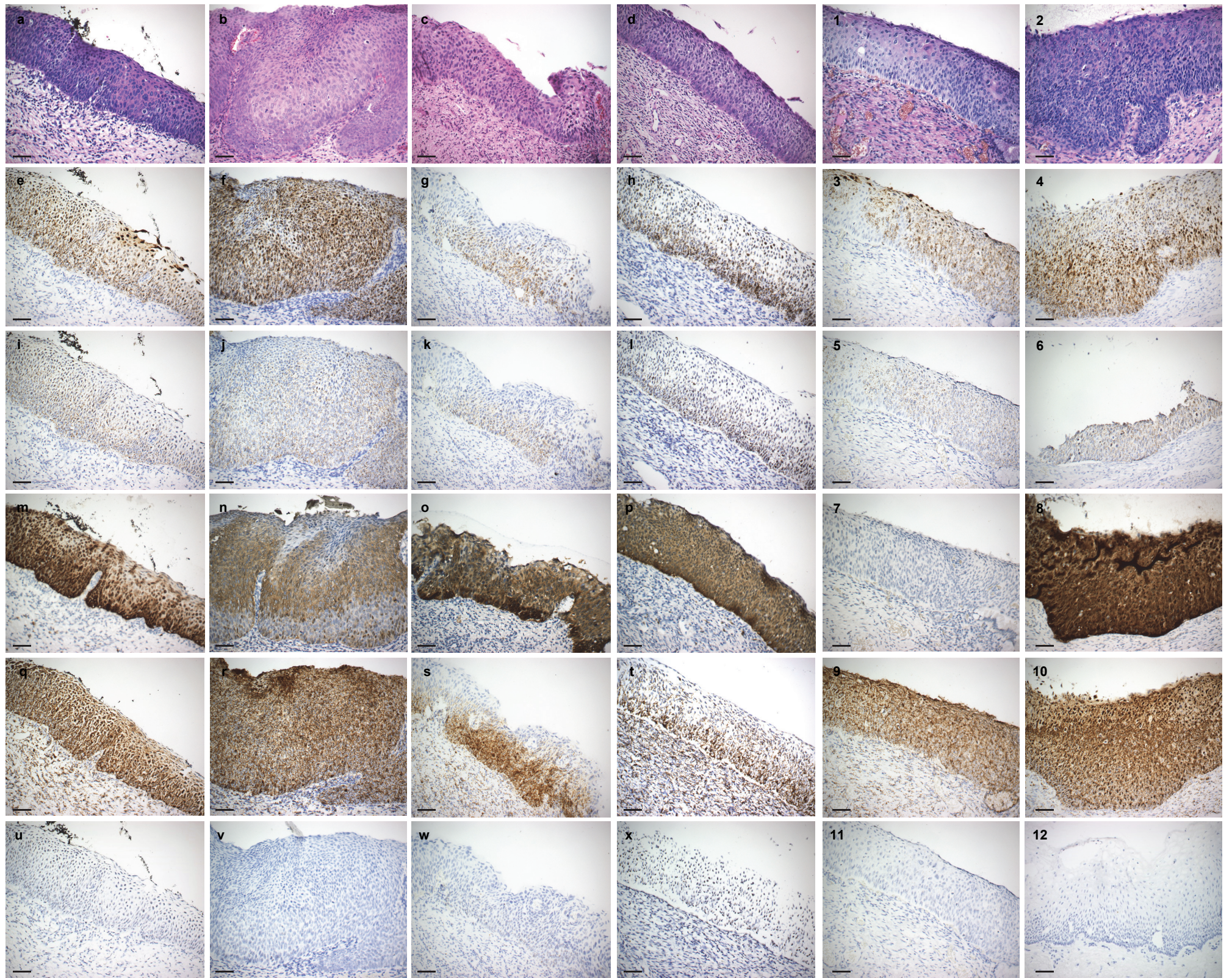

Supplement: Figure S3 — CIN 3 staining (expanded data). Top row: H&E; second row: HPV E6/E7 RNA CISH; third row: p16 RNA CISH; fourth row: p16INK4a IHC; fifth row: UBC RNA CISH; sixth row: E.Coli dap B RNA CISH. Columns a-d (see Figure 3 legend). Columns 1 & 2: HPV 16 positive lesions showing CIN 3 staining patterns; p16INK4a IHC staining was negative in one instance (S7). All images were originally taken using 20X objective lens. Scale bar: 50 µm. (PDF) [file pone.0091142.s003.pdf]

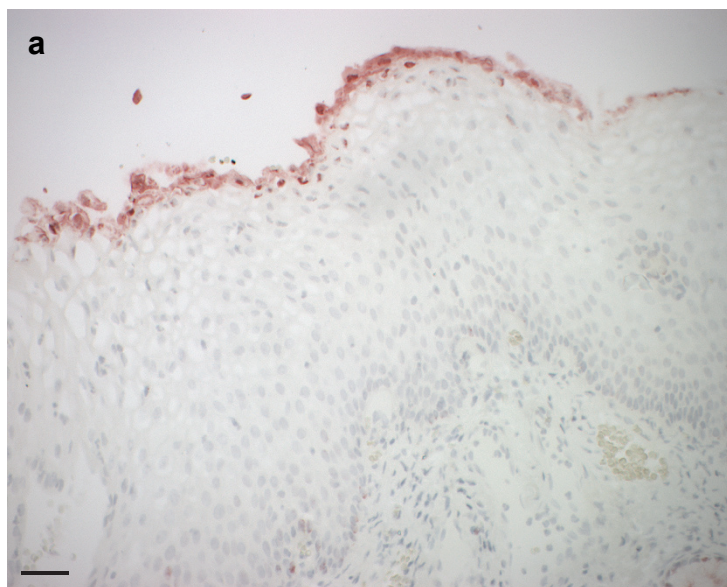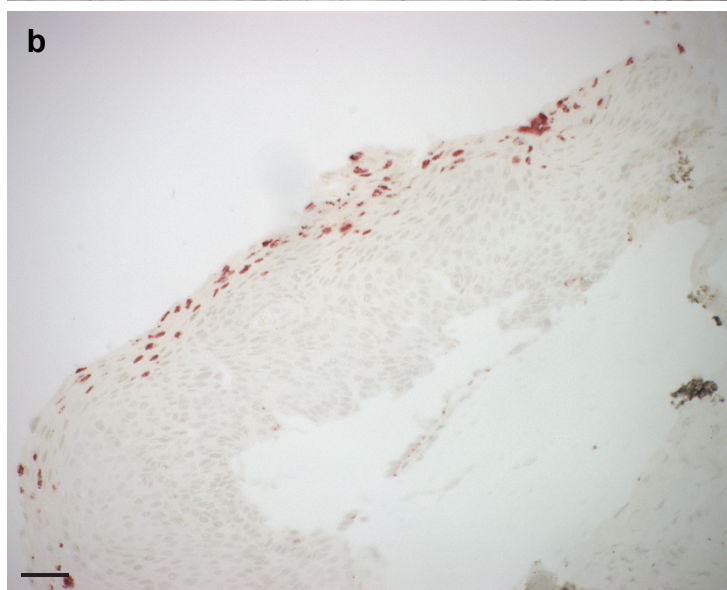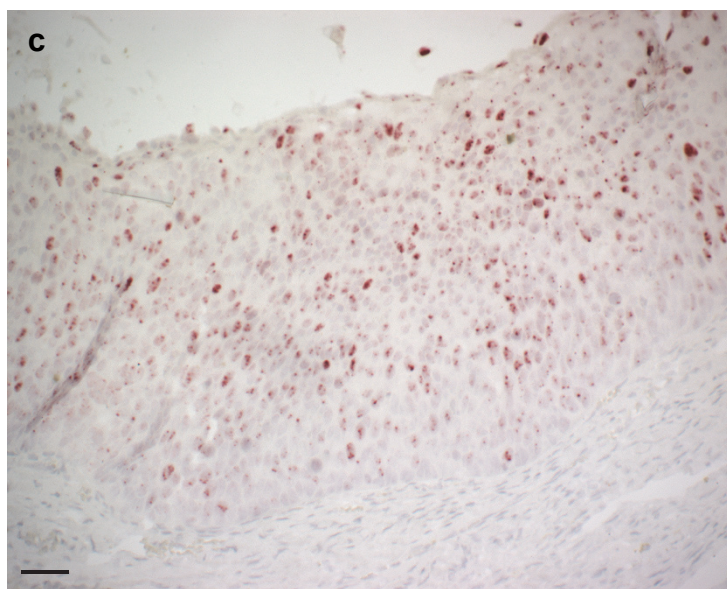

Supplement: Figure S4 — HPV DNA CISH. a. CIN 1 lesion superficial diffuse staining nuclei. b. CIN 2 lesion superficial diffuse staining nuclei. c. CIN 3 lesion showing diffuse and punctate signals through the thickness of the lesion. All images were originally taken using 20X objective lens. Scale bar: 50 µm. (PDF) [file pone.0091142.s004.pdf]

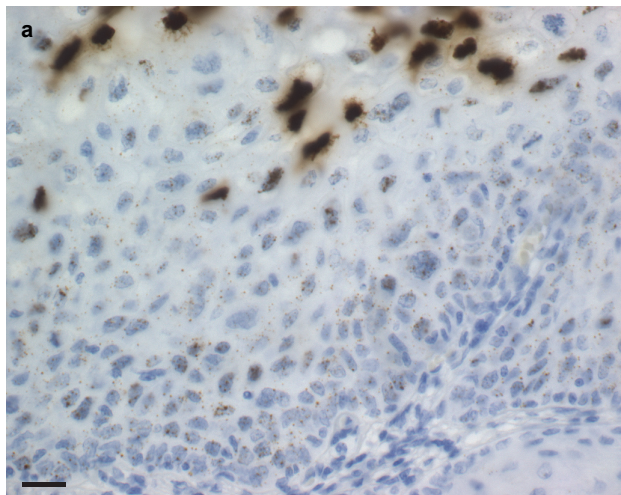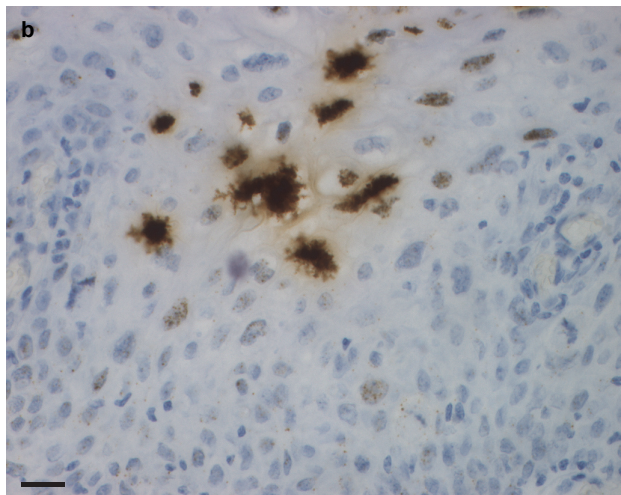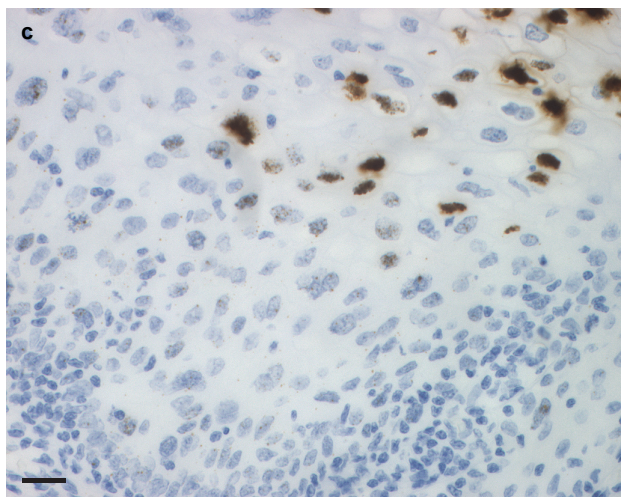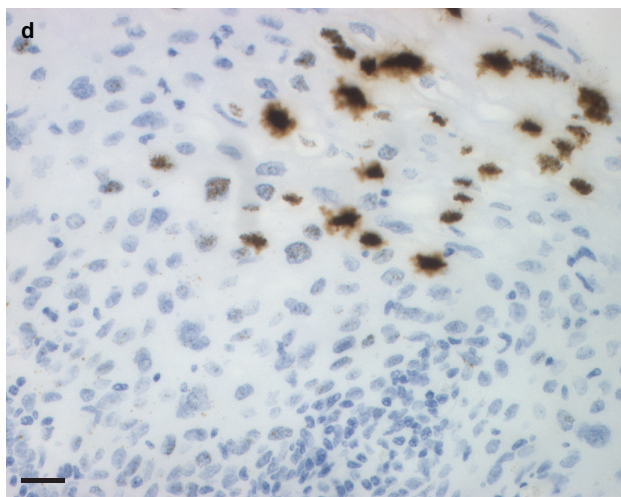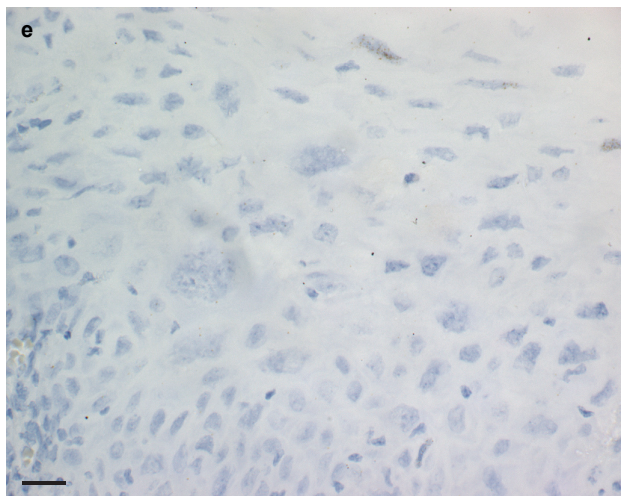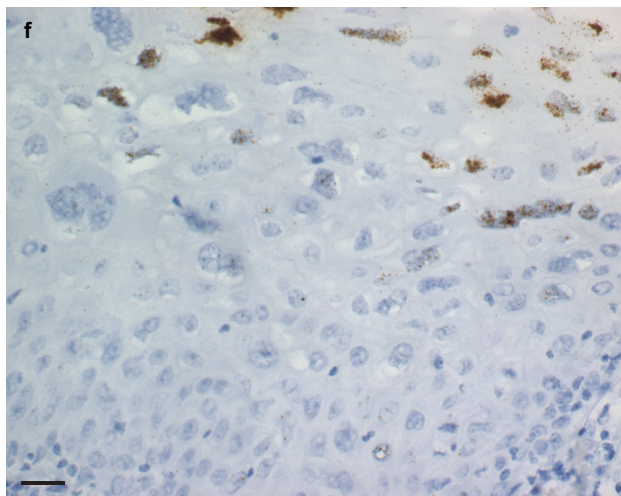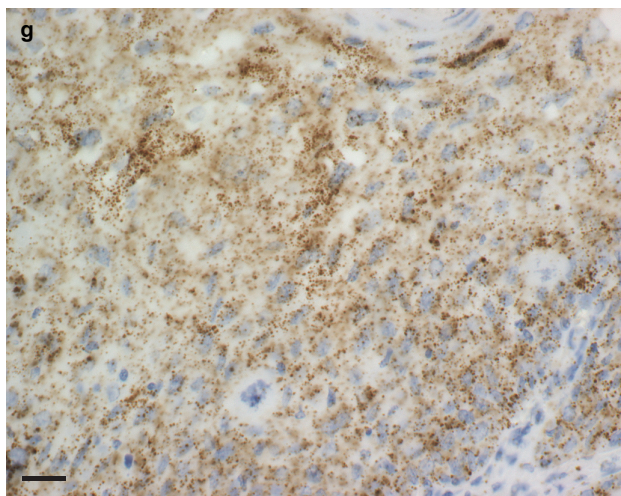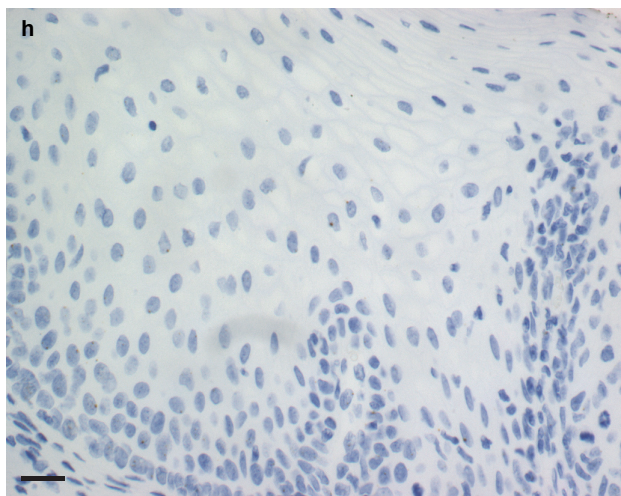

Supplement: Figure S5 — HPV E6/E7 RNA CISH control tests: productive phase HPV 16 infection. a. HPV diffuse nuclear staining signal patterns and fine nuclear and cytoplasmic dot-like signals after hybridization with antisense probe. b. HPV diffuse nuclear staining signal patterns after hybridization with sense-strand E6/E7 probe. These indicate that some diffuse nuclear staining may be HPV DNA. c. Reduced HPV diffuse nuclear staining signal patterns and absence of fine nuclear and cytoplasmic dot-like signals after hybridization with antisense probe on tissues pretreated with RNase A. These data suggest diffuse nuclear staining is (partially) due to hybridization with HPV DNA and that the fine nuclear and cytoplasmic signals are HPV RNA. d. HPV diffuse nuclear staining signal patterns after hybridization with sense-strand HPV probe on tissues pretreated with RNase A suggestive of hybridization with HPV DNA. e. Absence of HPV staining signal patterns after hybridization with anti-sense-strand HPV probe on tissues pretreated with DNase I confirming diffuse signal patterns involve probe hybridization with DNA. f. Reduced intensity diffuse HPV staining signal patterns after hybridization with sense-strand HPV probe on tissues pretreated with DNase I confirming diffuse signal patterns involve probe hybridization with DNA. Detected signals may be due to incomplete DNase digestion of abundant productive phase HPV DNA. g. & h. Abundant UBC RNA staining (g) eliminated (h) after tissue pretreatment with RNase A. All images were originally taken using 40X objective lens. Scale bar: 20 µm. (PDF) [file pone.0091142.s005.pdf]

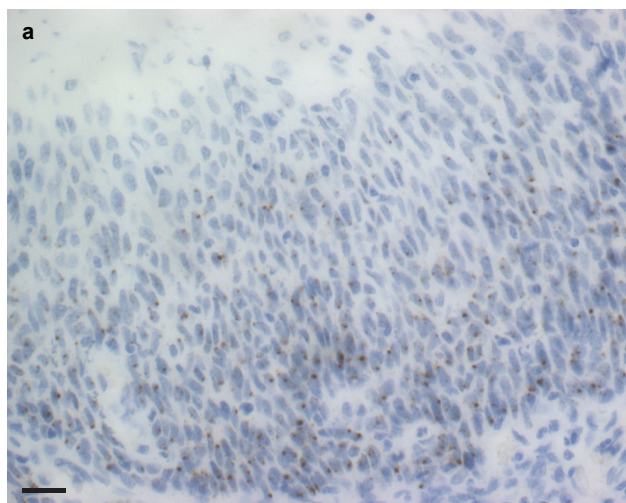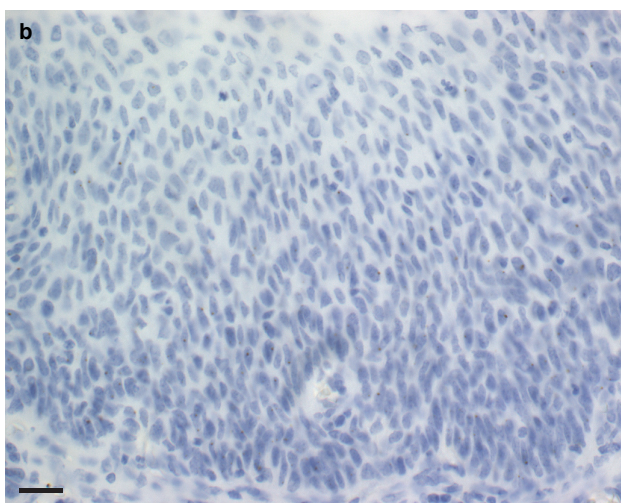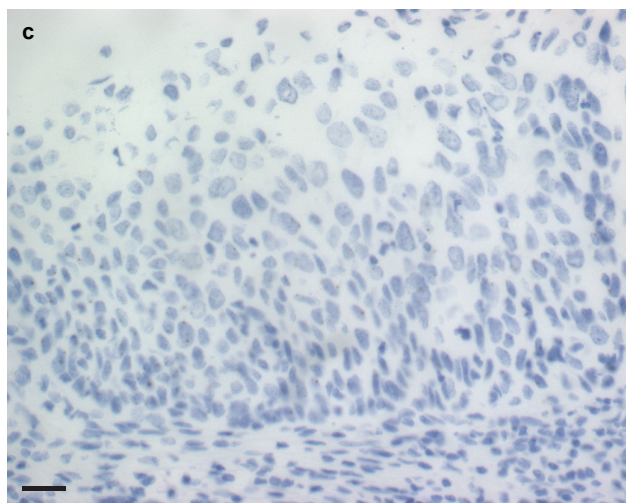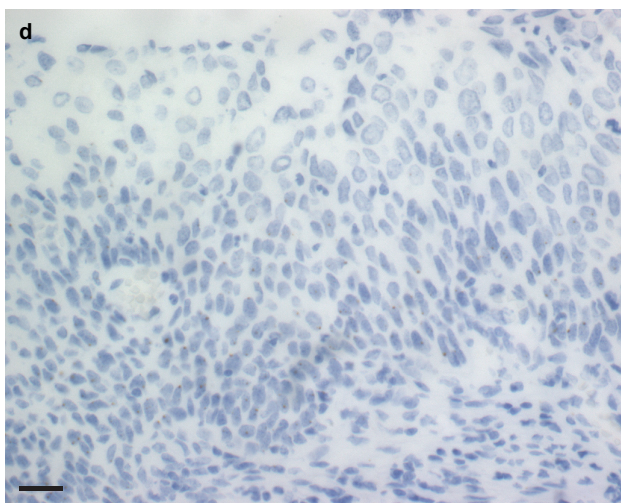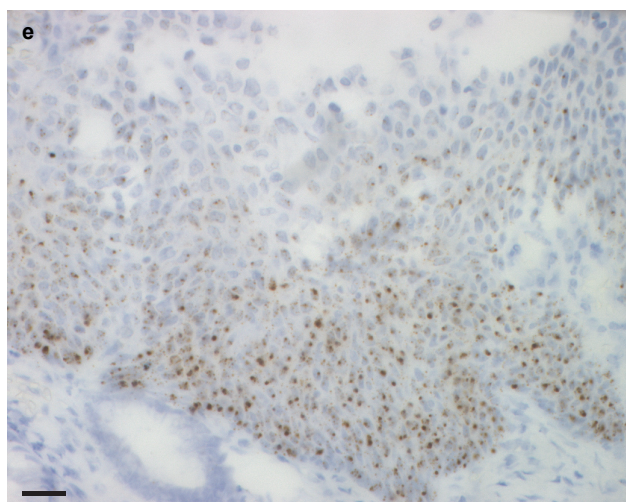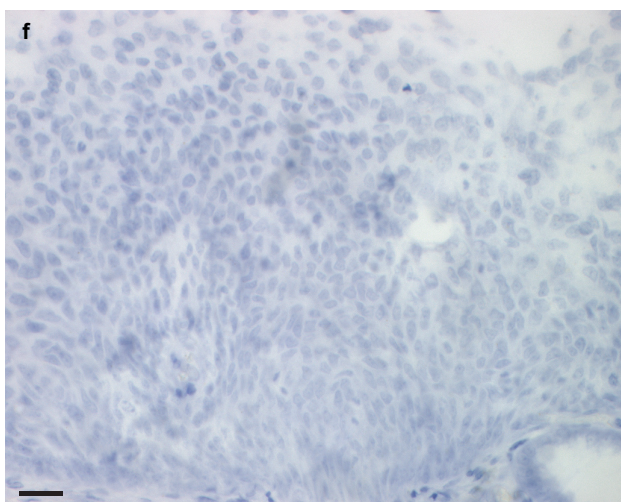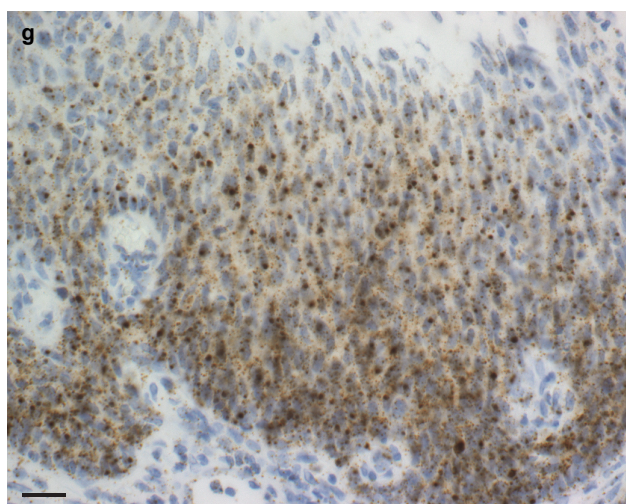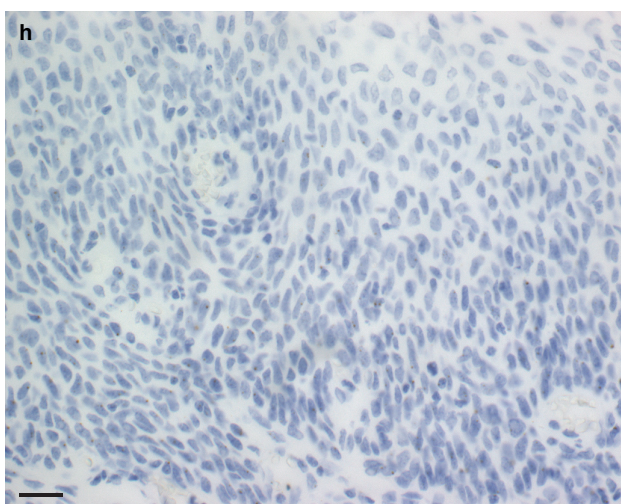

Supplement: Figure S6 — HPV E6/E7 RNA CISH control tests: transformative phase HPV 16 infection. a. HPV nuclear and cytoplasmic dot-like signals after hybridization with antisense probe. b. Absence of staining after hybridization with sense-strand HPV probe. This finding shows that the signals detected in (a) represent HPV RNA targets and not DNA. c. & d. Absence of any staining with anti-sense or sense-stand HPV probes on tissues pretreated with RNase A indicating the non-detection of HPV DNA targets. e. HPV positive staining after hybridization with antisense HPV probe on tissues pretreated with DNase I confirming signal patterns result from probe hybridization with RNA. f. Absence of staining in DNase I treated tissue hybridized with sense-strand HPV probe confirming that the anti-sense probes detect HPV E6/E7 RNA. g. & h. Abundant UBC RNA staining (g) eliminated (h) after tissue pretreatment with RNase A. All images were originally taken using 40X objective lens. Scale bar: 20 µm. (PDF) [file pone.0091142.s006.pdf]
